# Effective and efficient self-supervised masked model based on mixed feature training

**DOI:** 10.3389/fnbot.2025.1705970

**Published:** 2025-10-30

**Authors:** Qingjiu Kang, Feng Liu, Chunliu Cai

**Affiliations:** ^1^Institute of Artificial Intelligence, Guangzhou University, Guangzhou, Guangdong, China; ^2^Scientific Research Department, Guangzhou Preschool Teachers College, Guangzhou, China; ^3^Library, Guangdong University of Foreign Studies, Guangzhou, China

**Keywords:** masked image modeling, self-supervise learning, neuromorphic computing, Swin transformer, attention mechanism

## Abstract

Under the influence of Masked Language Modeling (MLM), Masked Image Modeling (MIM) employs an attention mechanism to perform masked training on images. However, processing a single image requires numerous iterations and substantial computational resources to reconstruct the masked regions, resulting in high computational complexity and significant time costs. To address this issue, we propose an Effective and Efficient self-supervised Masked model based on Mixed feature training (EESMM). First, we stack two images for encoding and input the fused features into the network, which not only reduces computational complexity but also enables the learning of more features. Second, during decoding, we obtain the decoding features corresponding to the original images based on the decoding features of the two input original images and the mixed images, and then construct a corresponding loss function to enhance feature representation. EESMM significantly reduces pre-training time without sacrificing accuracy, achieving 83% accuracy on ImageNet in just 363 h using four V100 GPUs–only one-tenth of the training time required by SimMIM. This validates that the method can substantially accelerate the pre-training process without noticeable performance degradation.

## 1 Introduction

Brain-inspired computing has provided a novel paradigm for addressing information perception and integration in complex environments. When processing multi-observation information, the human visual system does not simply perform pixel-level superposition of visual inputs (Liu et al., 2023). Instead, it employs a hierarchical, attention-guided fusion mechanism to efficiently extract redundant and complementary information, forming a stable and robust understanding of scenes. Inspired by this mechanism, this paper aims to solve the fusion problem of image sets (Chen et al., 2021; Grill et al., 2020). We observe that the key to the brain's information integration lies in the processes of selective attention and the formation of feature invariance. Specifically, the visual cortex enhances overlapping and consistent features (such as object edges and shapes) while suppressing inconsistent or noisy information, thereby constructing a more complete and reliable scene representation (Bao et al., 2021; Zhou et al., 2021).

Self-supervised learning is widely regarded as a brain-inspired paradigm, as it resembles the human brain's predictive coding mechanism that acquires knowledge by predicting missing information and refining internal representations without explicit supervision (Bao et al., 2021; He et al., 2022; Xie et al., 2022). Learning the intrinsic characteristics of images through attention mechanisms enables hierarchical visual processing models to autonomously acquire feature representations from data (Ren et al., 2025; Wang et al., 2023). Compared to traditional supervised learning, self-supervised learning relies on self-attention mechanisms to train on massive unlabeled data, significantly reducing annotation costs while enhancing the model's generalization capability in low-data scenarios. As a result, self-supervised learning has emerged as one of the mainstream approaches for tackling complex visual tasks (Chen et al., 2020; Dosovitskiy et al., 2020; Wei et al., 2022a).

In recent years, masked self-supervised learning methods have achieved remarkable progress in the visual domain, particularly Masked Image Modeling (MIM) models, which have demonstrated strong feature learning capabilities (Chen et al., 2020; Huang et al., 2022; Grill et al., 2020).

SimMIM, a representative MIM method, leverages a pyramid network structure and local-window self-attention mechanism to excel at capturing both detailed and global image features. However, as dataset sizes and image resolutions increase, the growth in data volume leads to longer training durations, and the computational cost of SimMIM rises dramatically (Wei et al., 2022a; Caron et al., 2021). Thus, reducing pretraining time and resource consumption while maintaining model performance has become a critical challenge in self-supervised learning (Huang et al., 2022; Li et al., 2023; Wei et al., 2022b; Kakogeorgiou et al., 2022).

GreenMIM (Huang et al., 2022) is an improvement on the traditional SimMIM method, introducing more efficient mask design and optimized model structure, reducing the amount of computation, thus improving the speed of SimMIM pre-training, and more suitable for large data sets and high-resolution images. Although GreenMIM further improves the computational efficiency of SimMIM, GreenMIM still relies on the single feature reconstruction method, that is, the mask design is used to optimize the network, so it does not fundamentally reduce the reconstruction computation of a single image (He et al., 2022; Baevski et al., 2022).

While most current self-supervised models prioritize reconstruction accuracy, they often overlook the impact of data redundancy on training efficiency, resulting in high computational costs during the pre-training phase. Existing research still lacks a universal solution that can significantly reduce pre-training complexity without altering the model architecture. Inspired by the visual system of the brain, which “integrates information through multiple observations,” this study addresses the research gap by proposing a lightweight masked modeling strategy from the perspective of input-level optimization. This is achieved through the superposition and separate reconstruction of multi-source images, effectively bridging this methodological void. we propose a novel self-supervised pre-training method called the Effectivee and Efficient Self-supervised Masked Model (EESMM). EESMM employs an innovative design that superimposes two different images at the pixel level to generate a mixed input incorporating features from both, while separately computing reconstruction losses for each original image during the decoding phase.

**Figure 1:**
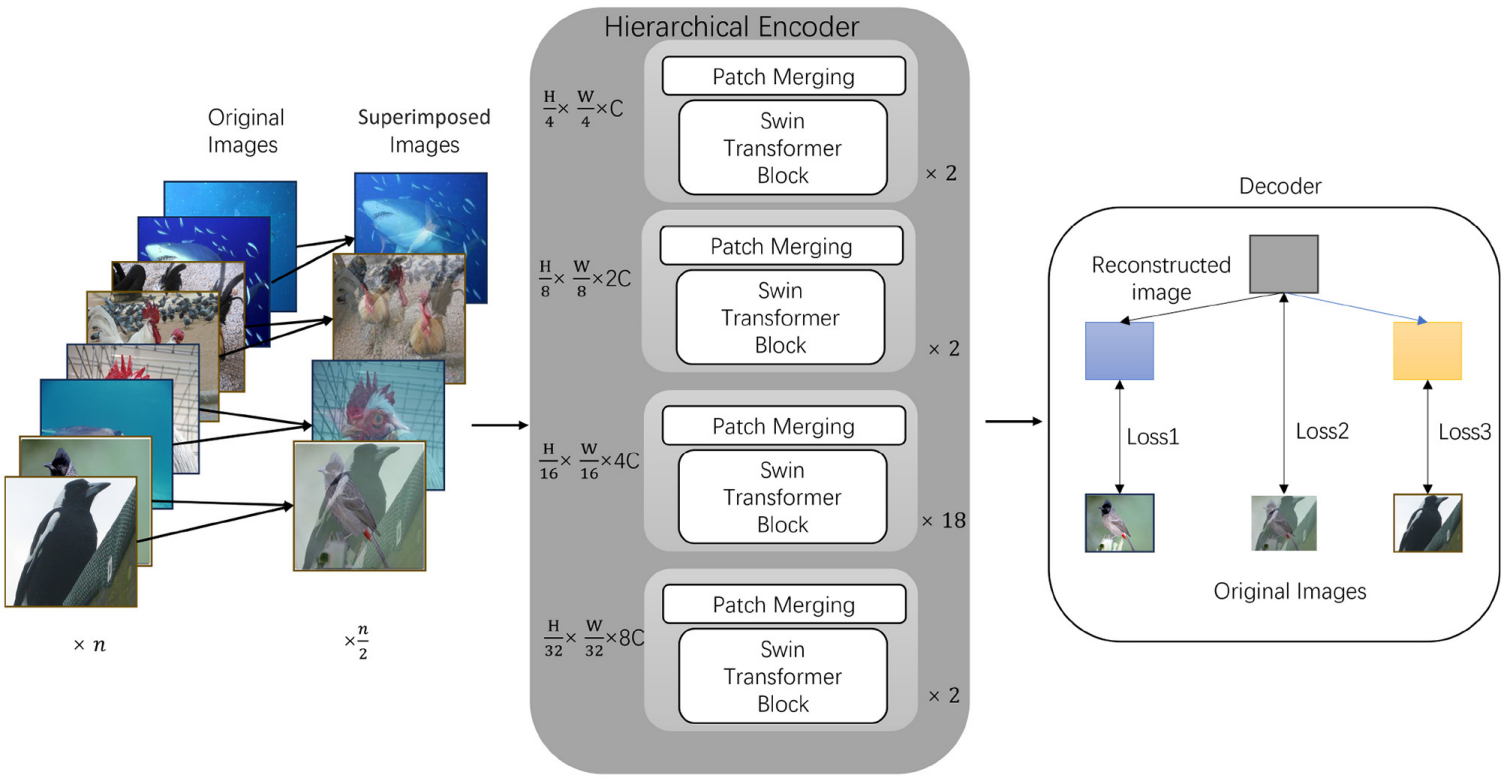
Model architecture of EESMM. We begin by combining the two original images into a single input through pixel-level fusion. The central part is a hierarchical encoder using Swin Transformer for feature extraction, where Patch Merging layers progressively reduce the feature map size. Finally, in the decoding and reconstruction stage, the reconstructed image is decomposed, and losses are calculated between the reconstructed parts and the original input images (Loss1, Loss2, and Loss3), as well as between the decomposed images and the pre-superimposed originals.

This mechanism enables the model to directly learn fused feature representations from the mixed input during training, rather than being limited to features from a single image. We present an overview of the network architecture in Figure 1, through the dual loss constraint in the decoder, it effectively prevents the loss of critical information during the fusion process, ensuring that the model accurately captures subtle differences in each image. Therefore, compared to self-supervised models such as SimMIM or GreenMIM, EESMM significantly improves pre-training efficiency while maintaining performance. The main contributions of this study can be summarized as follows:

1. We propose an effective and efficient mixed feature algorithm that incorporates image superposition techniques, significantly reducing training time and computational resource consumption while maintaining high model performance.

2. We design a decomposition-reconstruction mechanism for superimposed images and ensure effective feature learning of each input image by separately calculating losses, thereby mitigating information loss and enhancing feature representation capability.

3. Extensive experiments validate the effectiveness and applicability of EESMM. Experimental results demonstrate that our method outperforms most existing self-supervised learning methods on classification and visual tasks.

## 2 Related works

Inspired by BERT, the self-supervised learning approach Masked Image Modeling (MIM) trains models to predict or reconstruct randomly masked image patches or pixels, enabling the model to learn contextual information from the images. By forcing the model to understand both global and local features, MIM helps uncover the intrinsic structure of image content. This method demonstrates exceptional performance in hierarchical visual models such as Vision Transformer (ViT), enabling pretraining on unlabeled datasets and achieving excellent results in downstream vision tasks. The advantage of MIM lies in its ability to efficiently learn image features without requiring extensive labeled data, making it applicable to a variety of computer vision tasks such as classification, detection, and segmentation.

Devlin et al. (2019) introduced the BERT model to the vision domain. BEiT proposed a method for constructing a visual vocabulary by dividing input images into patches and applying a random masking strategy, allowing image patches to function similarly to tokens in NLP for prediction. BEiT uses Vision Transformer (ViT) as its backbone and encodes image patches into discrete identifiers via the visual vocabulary, enabling self-supervised pretraining. Experimental results demonstrated that pretrained BEiT models excelled in image classification tasks, significantly outperforming prior unsupervised learning methods (He et al., 2022).

Following BEiT, Masked Autoencoders (MAE) (He et al., 2022) introduced a simpler yet more scalable framework. MAE randomly masks parts of the input image, training the encoder using only the unmasked patches, which significantly reduces computational complexity. The decoder then reconstructs the masked patches based on the encoder's output. MAE's innovation lies in its minimalist architecture, enabling easy scalability to large datasets while delivering superior performance in downstream tasks like image classification and object detection.

SimMIM and iBOT (Xie et al., 2022; Zhou et al., 2021) are two influential MIM studies. SimMIM introduces a simple MIM pretraining framework, similar to MAE, where part of the image is randomly masked, and a simple decoder is used for reconstruction. iBOT extends BEiT by introducing an online tokenizer and self-supervised multi-task learning, improving the handling of image patches and performance in downstream tasks. Additionally, some studies explore cross-modal self-supervised learning, like Data2Vec (Baevski et al., 2022), a general SSL framework that applies to speech, vision, and language. Unlike traditional MIM methods, Data2Vec focuses on the applicability of a unified architecture across different data modalities.

In contrast to existing MIM methods, EESMM adopts a simplified masking and modeling strategy that significantly reduces pretraining time by half without substantial accuracy loss. Furthermore, by optimizing the structure of inputs and decoders, EESMM minimizes parameter count and computation, drastically cutting the time cost of pretraining.

## 3 Methodology

### 3.1 Preliminaries

Masked Image Modeling (MIM) is a self-supervised learning method that trains models to reconstruct masked regions of input images, capturing both global and local features. By applying a binary mask matrix **M** to the input image **I**, a masked image **I**_masked_ is created. The model's objective is to reconstruct the masked regions, producing $\hat{\text{I}}$. The mean squared error (MSE) loss function is used to measure the difference between the original image **I** and the reconstructed image $\hat{\text{I}}$, helping the model learn feature representations. This method is particularly effective in label-free scenarios, enhancing performance in tasks like image classification and object detection.

In this process, two input images, **I**_1_ and **I**_2_, are masked using binary masks, **M**_1_ and **M**_2_, which specify the masked regions. The masked images are obtained through element-wise multiplication:


(1) $$\begin{array}{l} {\text{I}}_{1,\text{masked}}={\text{I}}_{1}⊙{\text{M}}_{1}, \end{array}$$

(2) $$\begin{array}{l} {\text{I}}_{2,\text{masked}}={\text{I}}_{2}⊙{\text{M}}_{2} \end{array}$$

where ⊙ denotes element-wise multiplication. A mask value of 0 masks the pixel, while 1 retains it. This approach helps the model learn from incomplete information and improves its ability to recover original images, enhancing robustness and learning semantic features.

Hierarchical Vision Transformer (HViT) is an efficient visual transformer model that combines the hierarchical feature extraction mechanism of convolutional neural networks (CNNs) with the self-attention mechanism. It addresses the computational complexity of ViT in processing high-resolution images.

HViT mitigates this issue by introducing local self-attention mechanisms. The key idea is to compute self-attention within local windows after partitioning the image into patches, significantly reducing computation while preserving detailed information. The model progressively aggregates features layer by layer, transitioning from local to global feature extraction. At lower layers, HViT calculates feature dependencies between neighboring patches using local self-attention; at higher layers, global self-attention captures relationships across the entire image. This hierarchical design resembles CNN's feature pyramid, where the resolution of feature maps decreases layer by layer, but semantic information increases, achieving a balance between performance and efficiency.

Given an input image *X*∈ℝ^*H*×*W*×*C*^, HViT first divides it into patches of size *P*×*P*, flattens them into 1D vectors, and extracts neighborhood features using local self-attention. At higher layers, it employs global self-attention mechanisms to enhance the representation of the entire image. This hierarchical structure not only improves computational efficiency for high-resolution scenarios but also enhances the model's capability to understand global semantics.

### 3.2 Superposition-based masked image modeling

Despite the significant progress of prior masked image modeling (MIM) research in self-supervised visual representation pretraining, these methods often require extended pretraining time. This is primarily due to the necessity of processing large amounts of data, leading to considerable computational overhead and prolonged training time. However, in existing self-supervised learning models, large datasets are often indispensable, as data richness is key to learning effective visual features. To reduce data processing demands and shorten pretraining time, the EESMM model introduces a simple yet effective image superposition technique. For specific steps, please refer to Algorithm 1.

**Algorithm 1:** EESMM.

**input:** training dataset *B*_*train*_, test dataset *B*_*test*_, number of training cycles *E*
**output:** trained model *model*
# *L*: loss function.
# *x*∈ℝ^*P*×*h*×*w*×*c*^: *P* denotes the number of patches, *h*, *w* are the height and width of each patch, and *c* denotes the number of channels.
# num_patches∈ℝ^*N*×1^: number of patches per section, where *N* is the number of sections.
# mask_ratio: The ratio of mask patches.
# total_epoches: Number of pre-training cycles.
# epoch: Current cycle number.
Where *P* denotes the number of patches, *h* and *w* are the height and width of each patch respectively, and *c* denotes the number of channels.
initialize model *model* = *CreateEESMMModel*()
initialize optimizer *optimizer*
**for** per cycle *epoch* from 1 to *E* **do**
for each batch *batch* in *B*_*train*_ **do**
*X*←*batch*.*images*
$X_{composite}\leftarrow \frac{1}{n}\overset{n}{\underset{i=1}{\sum }}X_{i}$ // see Equation 3
{*X*_*masked*_, *M*}←*Mask*(*X*_*composite*_, ρ) // see Equation 1
*Z*←*model*.*encoder*(*X*_*masked*_)
$\hat{X}\leftarrow model.decoder\left(Z\right)$
$L\leftarrow L\left(\hat{X},X_{composite},M\right)$
*optimizer*.*zero*_*grad*()
*L*.*backward*()
*optimizer*.*step*()
**end for**
**end for**
**for** each batch *batch* in *B*_*test*_ **do**
*X*←*batch*.*images*
$X_{composite}\leftarrow \frac{1}{n}\overset{n}{\underset{i=1}{\sum }}X_{i}$
{*X*_*masked*_, *M*}←*Mask*(*X*_*composite*_, ρ)
*Z*←*model*.*encoder*(*X*_*masked*_)
$\hat{X}\leftarrow model.decoder\left(Z\right)$
$accuracy\leftarrow ComputeAccuracy\left(\hat{X},X_{composite}\right)$ {*accuracy*: accuracy of model prediction}
**end for**
**return** *model*

#### 3.2.1 Hybrid feature learning

**Figure 2:**
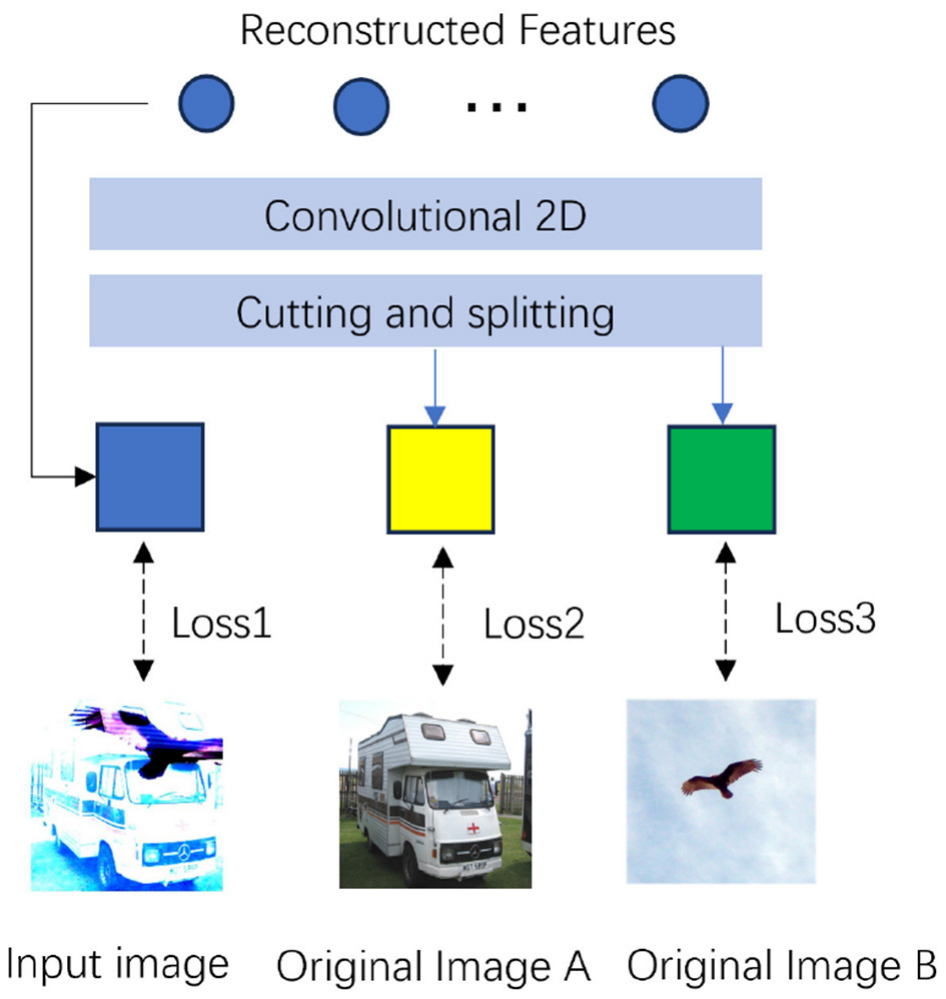
An illustration of the decoder construction, with the blue box representing the reconstructed image, and the yellow and green boxes representing the result of the decoder disassembling the superimposed image.

Hybrid feature learning refers to capturing multiple semantic features simultaneously by overlaying different image inputs, thereby improving the ability of the model to understand complex scenes. As shown in Figure 2, data compression is effectively achieved by combining two images to create a training sample. Specifically, two different images **I**_1_ and **I**_2_ are selected, weighted and combined to generate a new superimposed image,where the weight λ_1_ and the weight λ_2_ are fixed.: **I**_*t*_*extmixed* = λ_1_·**I**_1_+λ_2_·**I**_2_. We combine two occlusion images **I**_1, masked_ and **I**_2, masked_ processed as a mixed image **I**_mixed_ as input to the model. In other words, some of the information in the two images is integrated into a single representation, and the model only needs to process the superimposed image during training, thus greatly reducing the need to process large amounts of raw image data. The process of generating a mixed image is as follows:


(3) $$\begin{array}{l} {\text{I}}_{\text{mixed}}=\lambda_{1}\cdot \text{Rotate}\left({\text{I}}_{1,\text{masked}},\theta_{1}\right)\\ \;+\lambda_{1}\cdot \text{Flip}\left({\text{I}}_{1,\text{masked}},\text{horizontal}\right)\\ \;+\lambda_{2}\cdot \text{Scale}\left({\text{I}}_{2,\text{masked}},s_{2}\right)\\ \;+\lambda_{2}\cdot \text{Crop}\left({\text{I}}_{2,\text{masked}},r_{2}\right) \end{array}$$

During training, the model forces the model to learn the hybrid features in the hybrid image by reconstructing the two original images from the superimposed input images. The reconstruction process involves masking part of the image and calculating the loss function as the sum of the errors between the reconstructed image and the respective original images. It is ensured that each original image is recovered accurately.

This mechanism significantly improves the model's ability to process multi-source information, facilitating efficient feature extraction and reconstruction.

#### 3.2.2 Feature reconstruction and loss function

The decoder *f* takes the mixed image **I**_0_, created by overlaying two original images **I**_*a*_ and **I**_*b*_, as input and generates the reconstructed mixed image ${\hat{\text{I}}}_{c}$. By subtracting **I**_*a*_ and **I**_*b*_ from ${\hat{\text{I}}}_{c}$, the two reconstructed images ${\hat{\text{I}}}_{d}$ and ${\hat{\text{I}}}_{e}$ are obtained as follows:


(4) $$\begin{array}{l} {\hat{\text{I}}}_{c}=f\left({\text{I}}_{0}\right) \end{array}$$

(5) $$\begin{array}{l} {\hat{\text{I}}}_{d}={\hat{\text{I}}}_{c}-{\text{I}}_{b} \end{array}$$

(6) $$\begin{array}{l} {\hat{\text{I}}}_{e}={\hat{\text{I}}}_{c}-{\text{I}}_{a} \end{array}$$

The loss function is designed to measure the difference between the reconstructed and original images, guiding parameter updates. For each reconstructed result ${\hat{\text{I}}}_{1}$ and ${\hat{\text{I}}}_{2}$, the pixel-wise difference with the original images **I**_1_ and **I**_2_ is computed, using occlusion mask matrices **M**_1_ and **M**_2_ to specify which pixels contribute to the loss calculation. The loss function is defined as:


(7) $$\begin{array}{l} L_{\text{SP}}=\frac{1}{\left|{\text{M}}_{1}\right|}\underset{i,j}{\sum }m_{1,i,j}\cdot {\left({\text{I}}_{1,i,j}-{\hat{\text{I}}}_{1,i,j}\right)}^{2}\\ \;+\frac{1}{\left|{\text{M}}_{2}\right|}\underset{i,j}{\sum }m_{2,i,j}\cdot {\left({\text{I}}_{2,i,j}-{\hat{\text{I}}}_{2,i,j}\right)}^{2} \end{array}$$

Here, |**M**_1_| and |**M**_2_| represent the number of valid pixels in the mask matrices, i.e., the occluded pixels. The weighted summation of reconstruction errors ensures the model focuses on the occluded regions.

By extracting features from the blended image and reconstructing the original image, the model enhances its adaptability and robustness to recover the details of the original image, thus learning the features of the original image. This design encourages the model to leverage information from non-occluded regions for effective reconstruction, enhancing performance and maintaining high quality even with partial information.

## 4 Experiments

### 4.1 Training setup

#### 4.1.1 Pre-training setup

The input superimposed image with a size of 224 × 224 is first subjected to simple data enhancement (e.g., random cropping, horizontal flipping) and normalization. The encoder part is initialized with Swin Transformer (Liu et al., 2021) and a self-supervised pre-training strategy based on MAE (He et al., 2022). Specifically, we randomly mask 75% of the image patches and use a lightweight decoder with an embedding dimension of 512. The decoder takes as input the visible patch representations and masking tokens output by the encoder and appends them to the final stage of the encoder, which is used to learn the feature representations of the masked patches. Subsequently, the decoder predicts the normalized pixel values of the masked patches through a linear layer and splits the output of the superimposed image into two predicted images in ratio 1/2.

During the training process, the total training period of the model is 800 epochs, of which the first 100 epochs are the warm-up phase. The learning rate is gradually increased from 1e-6 to 1e-4, the base learning rate is set to 2e-4, the minimum learning rate is 1e-5, and the optimization is performed using the AdamW optimizer with a weight decay of 0.05 and a layer decay of 0.9. The batch size is 256. gradient accumulation is used in the training, and the epsilon value of the optimizer is set to 1e-6 to ensure the stability of the values.

#### 4.1.2 Fine-tuning setup

To fine-tune the model, we discard the decoder and directly append a 1,000-way fully connected layer to the average pooling output of the encoder as a classifier. The model is also optimized using the AdamW optimizer (Loshchilov and Hutter, 2017) for a total of 100 training epochs, with 20 warm-up epochs. The base/warm-up learning rates are 1.25 × 10^−4^/2.5 × 10^−7^, and a cosine annealing schedule (Loshchilov and Hutter, 2016) is used. Weight decay is set to 0.05, with layer-wise learning rate decay (Bao et al., 2021) set to 0.9/0.8/0.9. The stochastic depth ratios (Huang et al., 2016) for swwin-b/swwin-l/Twins-L are 0.2/0.3/0.2. Data augmentation is the same as in Bao et al. (2021).

### 4.2 Results

We compare EESMM with current state-of-the-art Masked Image Modeling methods in Table 1, including MAE and SimMIM. The evaluation metrics are mainly top-1 classification accuracy on the validation set and the pre-training time of the model, the experimental results are based on averages calculated from multiple independent runs. Note that all results of EESMM are obtained by supervised fine-tuning of the encoder through self-supervised pre-training without additional intermediate fine-tuning. In this experiment, we evaluate the performance of multiple models under different configurations to explore the impact of each factor on model accuracy. The experiments cover a variety of models using both ViT-B and Swin-B network architectures, with SimMIM192 achieving the best accuracy of 85.4% under the Swin-L network architecture. In contrast, EESMM achieved the lowest accuracy of 83.1% using the Swin-B network structure. In addition, the pre-training time for SimMIM224 was significantly longer at 3307 compared to 1.3 for MAE, and this difference may have affected the final accuracy of the model. Most of the other models have a training data volume of 86M or 88M and a training time of 800epoch or 1600epoch, but different data sources (e.g., DALL-E, HOG, RGB, and Feature) also have a significant effect on the accuracy. Overall, these results suggest that the choice of network structure, the length of pre-training time, and the data sources used have a significant impact on the final performance of the model.

**Table 1:** Comparison of different methods.

**Method**	**Backbone**	**Param.(M)**	**Epochs**	**Hours**	**Supervision**	**FT**
BEiT (Bao et al., 2021)	ViT-B	86	800	–	DALL-E	83.2
MAE (He et al., 2022)	ViT-B	86	1,600	2,069	RGB	83.6
MaskFeat (Wei et al., 2022a)	ViT-B	86	800	–	HOG	84.0
data2vec (Baevski et al., 2022)	ViT-B	86	800	–	Feature	84.2
iBOT (Zhou et al., 2021)	ViT-B	86	1,600	–	Momentum	84.0
EsViT (Li et al., 2021)	Swin-B/W14	87	300	–	Momentum	83.9
SimMIM224 (Xie et al., 2022)	ViT-B	86	800	3,307	RGB	83.8
SimMIM192 (Xie et al., 2022)	Swin-B	88	800	1,609	RGB	84.0
SimMIM192 (Xie et al., 2022)	Swin-L	197	800	2,821	RGB	85.4
CAE (Chen et al., 2024)	ViT-B	86	800	–	DALL-E	83.6
GreenMIM (Huang et al., 2022)	Swin-B	88	800	887	RGB	83.8
LoMaR (Chen et al., 2025)	ViT-B	90	300	–	RGB	83.3
Spikformer V2 (Zhou et al., 2024)	ViT-B	172	300	–	RGB	80.3
EESMM	Swin-B	88	800	363	RGB	83.1

Where hours represents the total time required for model training and FT represents the accuracy after fine-tune.

**Figure 3:**
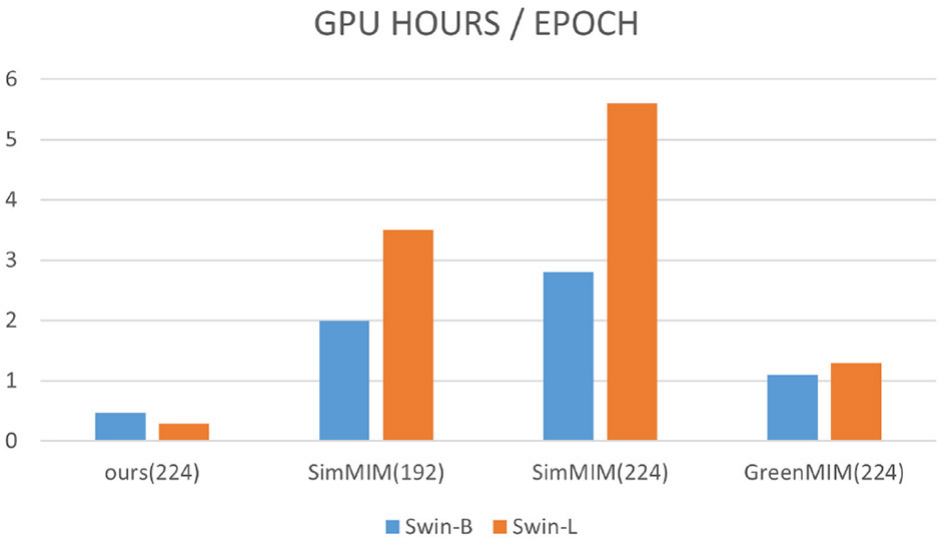
Comparison with other models in terms of efficiency. All methods use swing –b/swing –l trunks with a batch size of 2,048. The experiments for our method were performed on a machine with 2 V100 gpu, CUDA 11 and PyTorch 1.8, whereas the experiments for SimMIM required 2 or 4 machines.

Our method (224 input size) significantly improves computational efficiency with both Swin-B and Swin-L backbones. For example, with Swin-B, it requires only 0.47 GPU hours per epoch, compared to 2 and 2.8 h for SimMIM 192 and SimMIM 224, respectively. With Swin-L, our method takes just 0.29 GPU hours per epoch, while SimMIM 192 and SimMIM 224 take 3.5 and 5.6 GPU hours, respectively. This design reduces GPU consumption and computational overhead, maintaining efficient training and strong performance. Detailed comparisons are shown in Figure 3.

Table 2 compares the performance of various methods including EfficientNet-B5, EfficientNet-B7, ViT-B/16, ViT-L/16, Deit-B, CeiT, and our own model EESMM. All methods use ViT-B as the backbone network, while EESMM employs a Swin-B backbone. In terms of computational complexity, the FLOPs of EESMM is 11.3G, which falls between the values of other methods. On the CIFAR10 dataset, EESMM achieves the top accuracy of 99.1%, tying with Deit-B for the best performance. On the CIFAR100 dataset, EESMM attains an accuracy of 89.9%, which is an excellent result, second only to the 90.7% achieved by EfficientNet-B7.

**Table 2:** Performance comparison of different methods on the CIFAR-10 and CIFAR-100 fine-tuning benchmarks.

**Method**	**Backbone**	**FLOPs**	**CIFAR10**	**CIFAR100**
EfficientNet-B5 (Tan and Le, 2019)	ViT-B	10.3G	98.1	90.1
EfficientNet-B7 (Tan and Le, 2019)	ViT-B	37.3G	98.9	90.7
ViT-B/16 (Dosovitskiy et al., 2020)	ViT-B	18.7G	98.1	87.1
ViT-L/16 (Dosovitskiy et al., 2020)	ViT-B	65.8G	97.9	86.4
Deit-B (Touvron et al., 2021)	ViT-B	52.8G	99.1	89.8
CeiT (Yuan et al., 2021)	ViT-B	4.5G	99.0	89.8
EESMM	Swin-B	11.3G	99.1	89.9

Results are reported in classification accuracy (%). FLOPs indicate computational complexity.

### 4.3 Ablation study

In our ablation experiments, we use Swin-B as the backbone and reduce experimental overhead by setting the default input image size to 192 × 192. The window size is adjusted to 6 to accommodate the changed input dimensions. We use the ImageNet-1K image classification dataset for both pre-training and fine-tuning.

For self-supervised pre-training, we train the model for 100 epochs using the AdamW optimizer (Loshchilov and Hutter, 2017) with a cosine learning rate scheduler. The training hyperparameters are as follows: a batch size of 2048, a base learning rate of 8 × 10^−4^, a weight decay of 0.05, β_1_ = 0.9, and β_2_ = 0.999. A warm-up process is applied over the first 10 steps, utilizing light-data augmentation, including random resize cropping with a scaling range of [0.67, 1] and an aspect ratio range of [3/4, 4/3], followed by random flipping and color normalization.

The default settings for the EESMM component are as follows: a random masking strategy with a patch size of 32 × 32 and a masking ratio of 0.6, a linear prediction head targeting an output image size of 192 × 192, and an *L*_1_ loss for masked pixel prediction. Ablation studies are conducted by altering one setting at a time while keeping the others at their default values.

For fine-tuning, we also employ the AdamW optimizer with 100 epochs of training and a cosine learning rate scheduler, including a 10-epoch warm-up. The fine-tuning hyperparameters are as follows: a batch size of 2048, a base learning rate of 5 × 10^−3^, a weight decay of 0.05, β_1_ = 0.9, β_2_ = 0.999, a stochastic depth ratio of 0.1 (Huang et al., 2016), and hierarchical learning rate decay with a factor of 0.9. The data augmentation methods used include RandAugment (Cubuk et al., 2020), CutMix (Yun et al., 2019), label smoothing (Szegedy et al., 2016), and random erasing (Zhong et al., 2020).

For our approach, the evaluation was performed on a machine with four 32GB V100 gpu's, and for SimMIM, the evaluation was performed on 2 or 4 machines because it could not fit on one machine with the default batch size of the original paper (i.e., 2048). To verify the importance of the image overlay mechanism, we performed ablation experiments on ImageNet by removing the overlay module and using different masking ratios, respectively. The results of the experiments are shown in Table 3, where the pre-training time of the model increases significantly when the overlay module is removed, along with a slight decrease in the accuracy. We can see from the Figure 3 that for images of size 224^2^, SimMIM training is very slow and requires a lot of memory, and even though training with smaller images greatly reduces training time and memory consumption, it still lags far behind our method of training with images of size 224^2^. Specifically, for the same number of trainings, our method is comparable to the baseline performance using swing –b, with 2 × speedup and 60% memory reduction. We also observe that the improvement in efficiency grows as swing –L increases, e.g., a 2.7 × speedup compared to SimMIM192, which highlights the efficiency of our method with larger models.

**Table 3:** Comparison of EESMM with mainstream MIM models in terms of features and training time.

**Model**	**Adaptive VIT**	**Hot-swapping**	**Time (h)**
SimMIM	√	√	3,307
BEiT	√	×	–
MAE	×	×	2,069
GreenMIM	√	×	887
EESMM	√	√	363

**Figure 4:**
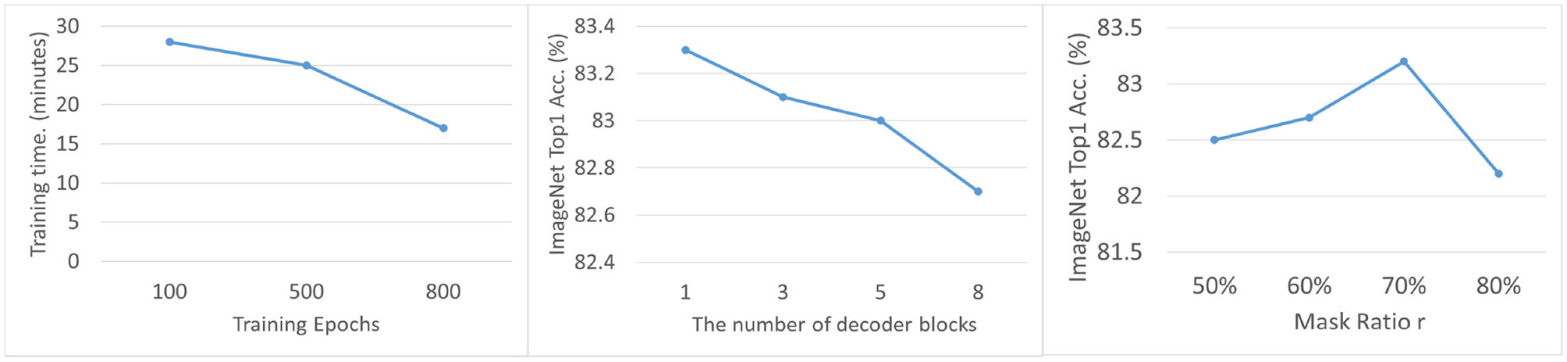
Ablation Studies of the mask ratio **(left)**, the decoder blocks **(middle)**, and the epochs **(right)**.

As shown in Figure 4 (right). When the training period is 100 epochs, the average training time per epoch is 28 minutes. As the number of training epochs increases to 500 epochs, the time for individual epochs decreases to 25 minutes, and as the number of training epochs further increases to 800 epochs, the training time for each epoch further decreases to 17 minutes. This shows that as the total training epochs increase, the training time for individual epochs shows a decreasing trend. This trend may be due to the gradual convergence of the model over a longer training period, or the fact that the model parameters become more efficiently tuned as the training progresses, leading to a reduction in the computational resources required for each epoch. We speculate that this phenomenon may be related to optimization techniques or efficient use of hardware resources during the training process, resulting in improved computational efficiency over more training cycles.

Moreover, we can see from the Figure 4 (left), as the Mask Ratio increases from 50 to 70%, the accuracy of the model gradually increases. When the Mask Ratio is 50%, the Top-1 accuracy is 82.5%; as the ratio increases to 60 and 70%, the accuracy increases to 82.7% and 83.1% respectively. However, when the Mask Ratio is further increased to 80%, the accuracy drops to 82.2%. This suggests that increasing the Mask Ratio within a certain range can improve the model performance, but too high a Mask Ratio may lead to a decrease in the accuracy, indicating the need to find the optimal balance of the Mask Ratio in model training.

## 5 Discussion and conclusion

In this paper, we present a hybrid and masked autoencoder (EESMM ) for efficient visual representation learning. Our EESMM uses a superimposed input created by blending two (or more) images with random masks and applies double reconstruction to recover the original two (or more) images from the superimposed hidden representation, which we further explore using Swin Transformer having a larger window size for efficient representation learning. One of the limitations of our algorithm is that it requires a batch masking scheme to achieve optimal efficiency. While this limitation has little impact on MIM pre-training, it restricts the application of our approach to a broader setup, e.g., the use of token sparification (Yin et al., 2021; Ramesh et al., 2021) trainingvit, which requires instantiated sparification. These applications are beyond the scope of the present work, and we will leave them reserved for future research. Finally, empirical results from multiple vision benchmarks show that EESMM can efficiently learn high-quality visual representations with a better FLOPs/performance tradeoff than previous MIM work. While this paper focuses on the visual domain, we hope that our work will inspire future work in other forms, such as text and audio.

## Data Availability

The datasets presented in this study can be found in online repositories. The names of the repository/repositories and accession number(s) can be found in the article/supplementary material.

## References

[B1] Baevski A. Hsu W.-N. Xu Q. Babu A. Gu J. Auli M.. (2022). “Data2vec: a general framework for self-supervised learning in speech, vision and language,” in International Conference on Machine Learning (Baltimore, MD: PMLR), 1298–1312.

[B2] Bao H. Dong L. Piao S. Wei F. (2021). Beit: bert pre-training of image transformers. arXiv [preprint]. arXiv:2106.08254. 10.48550/arXiv.2106.08254

[B3] Caron M. Touvron H. Misra I. Jégou H. Mairal J. Bojanowski P.. (2021). “Emerging properties in self-supervised vision transformers,” in Proceedings of the IEEE/CVF International Conference on Computer Vision (Montreal, QC: IEEE), 9650–9660. 10.1109/ICCV48922.2021.00951

[B4] Chen J. Khan F. F. Hu M. Sherif A. Ge Z. Li B.. (2025). “Local masked reconstruction for efficient self-supervised learning on high-resolution images,” in 2025 IEEE/CVF Winter Conference on Applications of Computer Vision (WACV) (Tucson, AZ), 8046–8056. 10.1109/WACV61041.2025.00781

[B5] Chen T. Kornblith S. Norouzi M. Hinton G. (2020). “A simple framework for contrastive learning of visual representations,” in International Conference on Machine Learning (Waikoloba, HI: PMLR), 1597–1607.

[B6] Chen X. Ding M. Wang X. Xin Y. Mo S. Wang Y.. (2024). Context autoencoder for self-supervised representation learning. Int. J. Comput. Vis. 132, 208–223. 10.1007/s11263-023-01852-4

[B7] Chen X. Xie S. He K. (2021). “An empirical study of training self-supervised vision transformers,” in Proceedings of the IEEE/CVF International Conference on Computer Vision (Montreal, QC: IEEE), 9640–9649. 10.1109/ICCV48922.2021.00950

[B8] Cubuk E. D. Zoph B. Shlens J. Le Q. V. (2020). “Randaugment: practical automated data augmentation with a reduced search space,” in Proceedings of the IEEE/CVF Conference on Computer Vision and Pattern Recognition Workshops (Seattle, WA: IEEE), 702–703. 10.1109/CVPRW50498.2020.00359

[B9] Devlin J. Chang M.-W. Lee K. Toutanova K. (2019). “Bert: pre-training of deep bidirectional transformers for language understanding,” in Proceedings of the 2019 Conference of the North American Chapter of the Association for Computational Linguistics: Human Language Technologies, Volume 1 (Long and Short Papers) (Minneapolis, MN), 4171–4186.

[B10] Dosovitskiy A. Beyer L. Kolesnikov A. Weissenborn D. Zhai X. Unterthiner T.. (2020). An image is worth 16x16 words: transformers for image recognition at scale. arXiv [preprint]. arXiv:2010.11929. 10.48550/arXiv:2010.11929

[B11] Grill J.-B. Strub F. Altché F. Tallec C. Richemond P. Buchatskaya E.. (2020). Bootstrap your own latent-a new approach to self-supervised learning. Adv. Neural Inf. Process. Syst. 33, 21271–21284. 10.48550/arXiv.2006.07733

[B12] He K. Chen X. Xie S. Li Y. Dollár P. Girshick R. (2022). “Masked autoencoders are scalable vision learners,” in Proceedings of the IEEE/CVF Conference on Computer Vision and Pattern Recognition (New Orleans, LA: IEEE), 16000–16009. 10.1109/CVPR52688.2022.01553

[B13] Huang G. Sun Y. Liu Z. Sedra D. Weinberger K. Q. (2016). “Deep networks with stochastic depth,” in European Conference on Computer Vision (Cham: Springer), 646–661. 10.1007/978-3-319-46493-0_39

[B14] Huang L. You S. Zheng M. Wang F. Qian C. Yamasaki T.. (2022). Green hierarchical vision transformer for masked image modeling. Adv. Neural Inf. Process. Syst. 35, 19997–20010.

[B15] Kakogeorgiou I. Gidaris S. Psomas B. Avrithis Y. Bursuc A. Karantzalos K.. (2022). “What to hide from your students: attention-guided masked image modeling,” in European Conference on Computer Vision (Cham: Springer), 300–318. 10.1007/978-3-031-20056-4_18

[B16] Li C. Yang J. Zhang P. Gao M. Xiao B. Dai X.. (2021). Efficient self-supervised vision transformers for representation learning. arXiv [preprint]. arXiv:2106.09785. 10.48550/arXiv:2106.09785

[B17] Li T. Chang H. Mishra S. Zhang H. Katabi D. Krishnan D.. (2023). “Mage: masked generative encoder to unify representation learning and image synthesis,” in Proceedings of the IEEE/CVF Conference on Computer Vision and Pattern Recognition (Vancouver, BC: IEEE), 2142–2152. 10.1109/CVPR52729.2023.00213

[B18] Liu Z. Gui J. Luo H. (2023). Good helper is around you: attention-driven masked image modeling. Proc. AAAI Conf. Artif. Intell. 37, 1799–1807. 10.1609/aaai.v37i2.25269

[B19] Liu Z. Lin Y. Cao Y. Hu H. Wei Y. Zhang Z.. (2021). “Swin transformer: Hierarchical vision transformer using shifted windows,” in Proceedings of the IEEE/CVF International Conference on Computer Vision (Montreal, QC: IEEE), 10012–10022. 10.1109/ICCV48922.2021.00986

[B20] Loshchilov I. Hutter F. (2016). Sgdr: Stochastic gradient descent with warm restarts. arXiv [preprint]. arXiv:1608.03983. 10.48550/arXiv.1608.03983

[B21] Loshchilov I. Hutter F. (2017). Decoupled weight decay regularization. arXiv [preprint]. arXiv:1711.05101. 10.48550/arXiv.1711.05101

[B22] Ramesh A. Pavlov M. Goh G. Gray S. Voss C. Radford A.. (2021). “Zero-shot text-to-image generation,” in International Conference on Machine Learning (PMLR), 8821–8831.

[B23] Ren S. Wei F. Zhang S. A. Z. Hu H. (2025). “Deepmim: deep supervision for masked image modeling,” in 2025 IEEE/CVF Winter Conference on Applications of Computer Vision (WACV) (Tucson, AZ: IEEE), 879–888. 10.1109/WACV61041.2025.00095

[B24] Szegedy C. Vanhoucke V. Ioffe S. Shlens J. Wojna Z. (2016). “Rethinking the inception architecture for computer vision,” in Proceedings of the IEEE Conference on Computer Vision and Pattern Recognition (Las Vegas, NV: IEEE), 2818–2826. 10.1109/CVPR.2016.308

[B25] Tan M. Le Q. (2019). “Efficientnet: rethinking model scaling for convolutional neural networks,” in International Conference on Machine Learning (Long Beach, CA: PMLR), 6105–6114.

[B26] Touvron H. Cord M. Douze M. Massa F. Sablayrolles A. Jégou H. (2021). “Training data-efficient image transformers & distillation through attention,” in International Conference on Machine Learning (PMLR), 10347–10357.

[B27] Wang H. Tang Y. Wang Y. Guo J. Deng Z.-H. Han K.. (2023). “Masked image modeling with local multi-scale reconstruction,” in Proceedings of the IEEE/CVF Conference on Computer Vision and Pattern Recognition (Vancouver, BC: IEEE),2122–2131. 10.1109/CVPR52729.2023.00211

[B28] Wei C. Fan H. Xie S. Wu C.-Y. Yuille A. Feichtenhofer C.. (2022a). “Masked feature prediction for self-supervised visual pre-training,” in Proceedings of the IEEE/CVF Conference on Computer Vision and Pattern Recognition (New Orleans, LA: IEEE), 14668–14678. 10.1109/CVPR52688.2022.01426

[B29] Wei L. Xie L. Zhou W. Li H. Tian Q. (2022b). “MVP: multimodality-guided visual pre-training,” in European Conference on Computer Vision (Cham: Springer), 337–353. 10.1007/978-3-031-20056-4_20

[B30] Xie Z. Zhang Z. Cao Y. Lin Y. Bao J. Yao Z.. (2022). “SimMIM: a simple framework for masked image modeling,” in Proceedings of the IEEE/CVF Conference on Computer Vision and Pattern Recognition (New Orleans, LA: IEEE), 9653–9663. 10.1109/CVPR52688.2022.00943

[B31] Yin H. Vahdat A. Alvarez J. Mallya A. Kautz J. Molchanov P.. (2021). Adavit: Adaptive tokens for efficient vision transformer. arXiv [preprint]. arXiv:2112.07658. 10.48550/arXiv.2112.07658

[B32] Yuan K. Guo S. Liu Z. Zhou A. Yu F. Wu W.. (2021). “Incorporating convolution designs into visual transformers,” in Proceedings of the IEEE/CVF International Conference on Computer Vision (Montreal, QC: IEEE), 579–588. 10.1109/ICCV48922.2021.00062

[B33] Yun S. Han D. Oh S. J. Chun S. Choe J. Yoo Y.. (2019). “Cutmix: regularization strategy to train strong classifiers with localizable features,” in Proceedings of the IEEE/CVF International Conference on Computer Vision (Seoul: IEEEE), 6023–6032. 10.1109/ICCV.2019.00612

[B34] Zhong Z. Zheng L. Kang G. Li S. Yang Y. (2020). Random erasing data augmentation. Proc. AAAI Conf. Artif. Intell. 34, 13001–13008. 10.1609/aaai.v34i07.7000

[B35] Zhou J. Wei C. Wang H. Shen W. Xie C. Yuille A.. (2021). IBOT: image bert pre-training with online tokenizer. arXiv [preprint]. arXiv:2111.07832. 10.48550/arXiv.2111.07832

[B36] Zhou Z. Che K. Fang W. Tian K. Zhu Y. Yan S.. (2024). Spikformer v2: join the high accuracy club on imagenet with an SNN ticket. arXiv [preprint]. arXiv:2401.02020. 10.48550/arXiv.2401.02020

